# Indoor heat and subjective well-being in older adults

**DOI:** 10.1093/geroni/igaf122.3287

**Published:** 2025-12-31

**Authors:** Amir Baniassadi, Wanting Yu, Thomas Travison, Lewis Lipsitz, Brad Manor

**Affiliations:** Hebrew SeniorLife, Boston, Massachusetts, United States; Hebrew SeniorLife, Boston, Massachusetts, United States; Hebrew SeniorLife & Harvard Medical School, Hebrew SeniorLife, Massachusetts, United States; Hebrew Senior Life, Boston, Massachusetts, United States; Hebrew SeniorLife/Harvard Medical School, Boston, Massachusetts, United States

## Abstract

Many older adults frequently experience cognitive difficulties, depressed mood, and discomfort in daily life, yet the impact of their home temperature fluctuations on these important subjective outcomes remains understudied. We conducted a 12-month observational study involving 47 community-dwelling older adults in Boston, MA. Participants’ home ambient temperatures were continuously monitored, and they completed twice-daily surveys assessing self-reported experience of cognitive difficulties (“difficulty keeping attention”), thermal discomfort (“feeling hot”), and mood (“feeling down or depressed”). We collected 17,201 Ecological Momentary Assessments, which were time-synced with home temperature sensor data. The mean indoor temperature at the time of assessments was 22.46 ± 2.4 °C. Using Generalized Additive Models (GAMs) controlling for humidity and other relevant covariates, we found that the likelihood of experiencing difficulty keeping attention and the likelihood of feeling hot were each lowest between 20–24 °C. Above this optimal range, every 4 °C increase in ambient temperature corresponded to approximately a 10-fold increase in the likelihood of reporting feeling hot (P < 0.001) and doubled the likelihood of experiencing difficulty keeping attention (P < 0.001). Although temperature showed a statistically significant relationship with negative mood, the magnitude of this effect was negligible and of limited practical significance. Future studies might benefit from evaluating mood using more sensitive measures. Based on our findings, indoor heat fluctuations appear to impact subjective well-being and cognitive difficulties among older adults. A climate- and location- specific optimization of indoor ambient temperature may therefore protect cognitive functioning and enhance comfort in older populations.

